# (*E*)-3-{4,6-Dimeth­oxy-2-[(*E*)-4-meth­oxy­styr­yl]-3-methyl­phen­yl}-1-(2-hy­droxy-5-meth­oxy­phen­yl)prop-2-en-1-one

**DOI:** 10.1107/S2414314620007920

**Published:** 2020-06-16

**Authors:** Miri Yoo, Dongsoo Koh

**Affiliations:** a Department of Applied Chemistry, Dongduk Women’s University, Seoul 136-714, Republic of Korea; University of Aberdeen, Scotland

**Keywords:** crystal structure, chalcone, resveratrol, C—H⋯O hydrogen bonds

## Abstract

In the title compound, C_28_H_28_O_6_, the benzene rings in the resveratrol moiety are connected by a *trans* C=C double bond, and the hydroxyl group containing a benzene ring and the central benzene ring are linked through a C=(O)—C=C (enone) moiety to form a chalcone unit. An intra­molecular O—H⋯O hydrogen bond generates an *S*(6) ring motif. In the crystal, pairs of C—H⋯O hydrogen bonds generate dimers and additional weak C—H⋯O inter­actions link the dimers into chains propagating along the *b*-axis direction.

## Abstract 2

The crystal structure of a pharmacophore hybrid of chalcone and resveratrol is reported.

## Graphical abstract

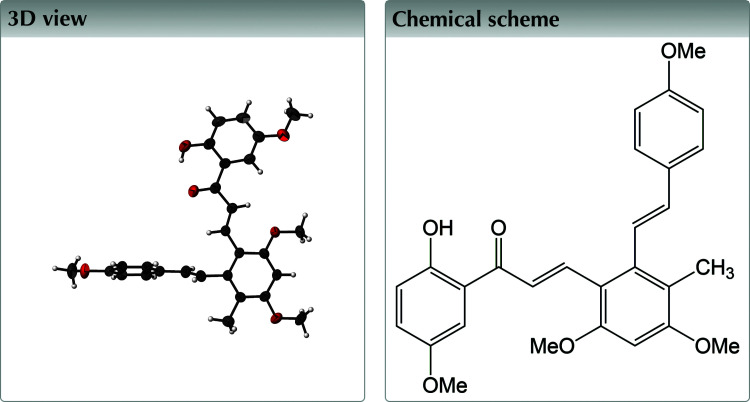

## Structure description

Resveratrol is a secondary metabolite of plants. It has been shown to have phytoalexin abilities, which can be considered to be a self-defense system in the plants to protect from pathogen infections (Timperio *et al.*, 2012 ▸). Chalcones, which are another essential secondary metabolites of plants, have been shown to possess diverse biological activities in our previous studies (Gil *et al.*, 2018 ▸; Lee *et al.*, 2016 ▸). The title compound, (I), was designed to combine the chalcone and resveratrol units in order to explore its biological activities (Zhuang *et al.*, 2017 ▸).

**Figure 1:**
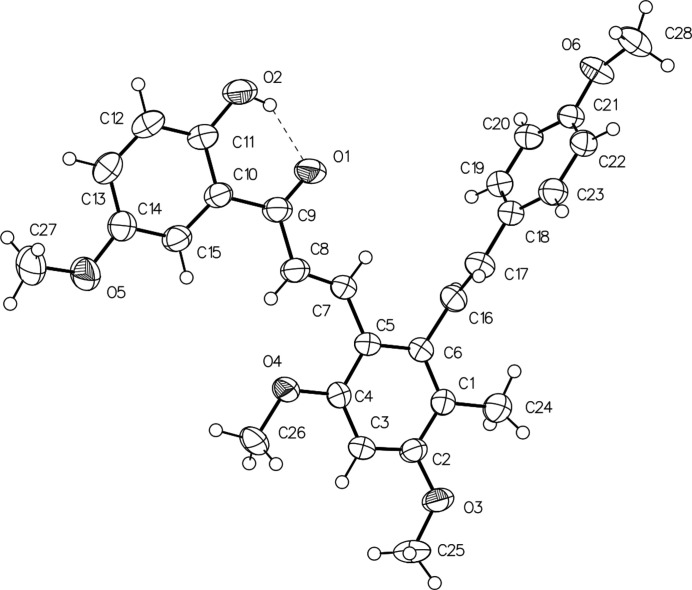
The mol­ecular structure of (I) with displacement ellipsoids drawn at the 30% probability level.

The mol­ecular structure of (I) is shown in Fig. 1 ▸. The benzene ring (C1–C6) in the centre of the mol­ecule participates in chalcone formation through the –C7=C8—C9=(O1)– (enone) linkage and participates in the resveratrol unit through the C16=C17 double bond. In the resveratrol unit, the dihedral angle between the central benzene ring (C1–C6) and the C18–C23 benzene ring is 84.8 (2)°, which indicates that the rings are almost orthogonal to each other. On the other hand, in the chalcone unit, the dihedral angle formed by the central benzene ring and the C15–C15 benzene ring is 9.34 (1)°, which make the two rings close to coplanar. There are four meth­oxy groups attached to carbon atoms C2, C4, C14 and C21 of the benzene rings in (I). The C26 atom of the meth­oxy group at C2 is almost co-planar with the benzene ring [C3—C4—O4—C26 = 0.8 (3)°], whereas atoms C25, C27 and C28 of the meth­oxy groups at C2, C14 and C21, respectively, are slightly twisted from the corresponding ring planes [C3—C2—O3—C25 = −5.3 (3); C13—C14—O5—C27 = −10.2 (4); C22—C21—O6—C28 = −10.6 (3)°]. The hydroxyl group attached to the C10–C15 benzene ring forms an intra­molecular O2—H2⋯O1 hydrogen bond with carbonyl O atom of the chalcone unit (Table 1 ▸).

**Figure 2:**
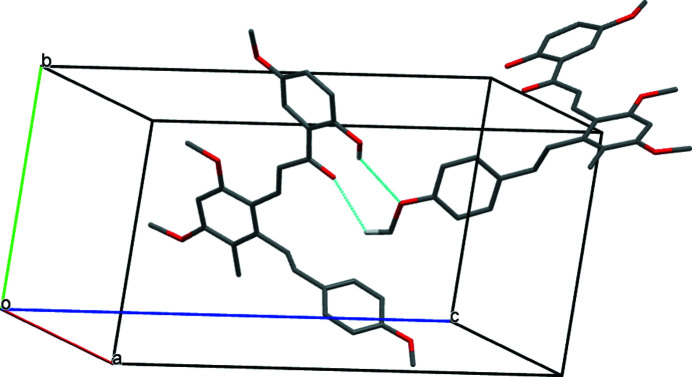
A view of a dimer linked by pairwise C—H⋯O hydrogen bonds (dashed lines) in the crystal structure of (I). For clarity only those H atoms involved in hydrogen bonding are shown.

**Figure 3:**
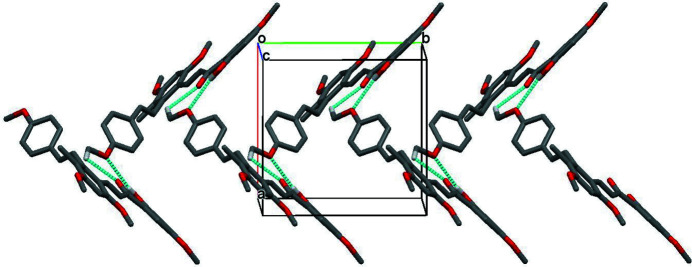
Part of the crystal structure of (I) with hydrogen bonds (blue dashed lines) shown. For clarity only those H atoms involved in hydrogen bonding are shown.

In the crystal, pairs of C—H⋯O hydrogen bonds generate inversion dimers (Table 1 ▸, Fig. 2 ▸) and another pair of C—H⋯O hydrogen bonds links the dimers into chains propagating along [010] (Fig. 3 ▸). Given their H⋯O lengths of greater than 2.60 Å, these hydrogen bonds are presumably very weak.

## Synthesis and crystallization

**Figure 4:**
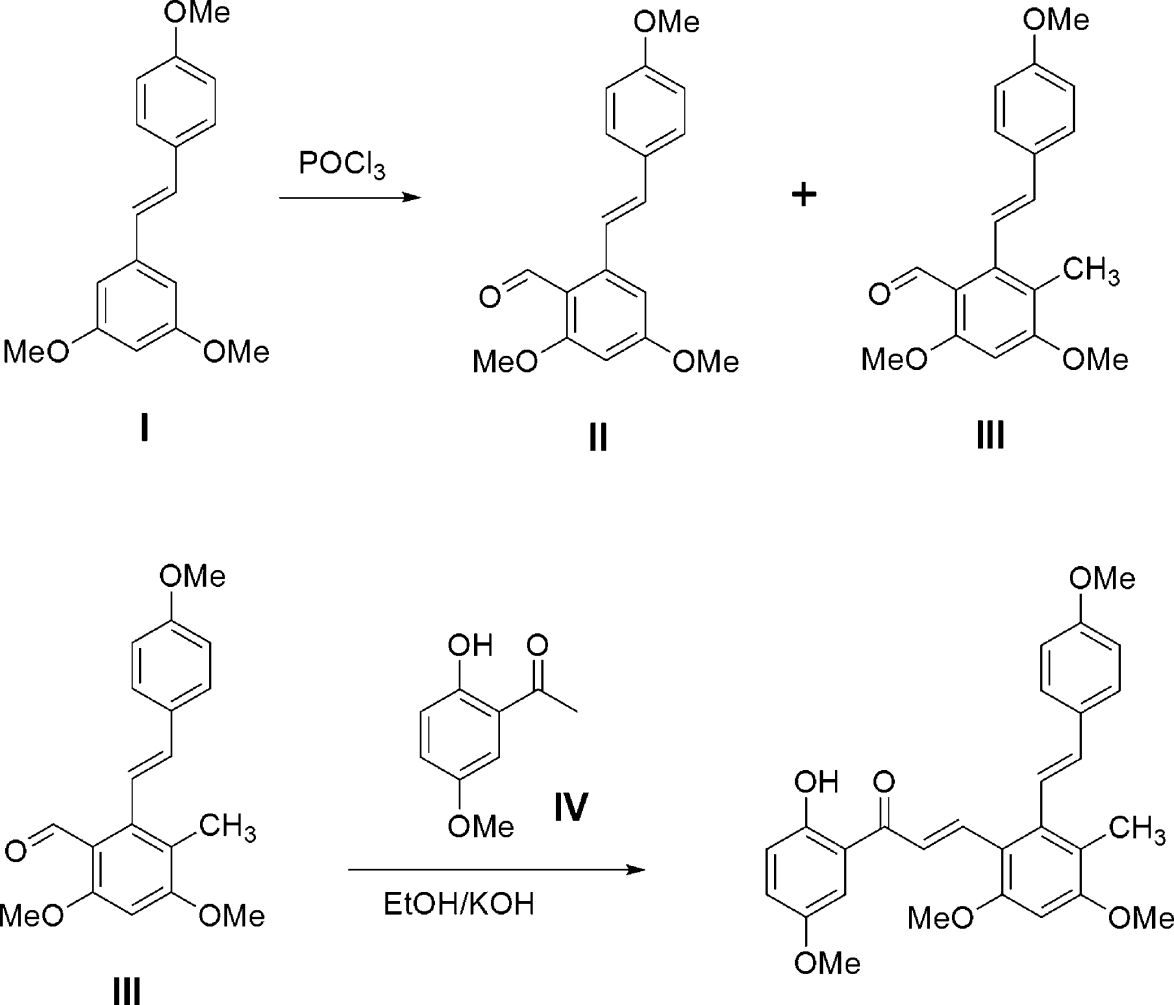
Synthetic scheme for the preparation of (I).

The synthetic scheme for the preparation of the title compound is shown in Fig. 4 ▸. Using the previously reported method (Shin *et al.* 2019 ▸), the resveratrol aldehyde inter­mediates **II** and **III** were prepared in 30% and 15% yields, respectively, from trimeth­oxy resveratrol (**I**). The methyl­ated resveratrol aldehyde inter­mediate **III** was reacted with 2-hy­droxy-5-meth­oxy aceto­phenone (**IV**) under Claisen–Schmidt condensation conditions to give the desired title compound. Recrystallization of the final adduct from ethanol solution gave crystals of the title compound in the form of orange blocks.

## Refinement

Crystal data, data collection and structure refinement details are summarized in Table 2 ▸.

## Supplementary Material

Crystal structure: contains datablock(s) I, global. DOI: 10.1107/S2414314620007920/hb4350sup1.cif

Structure factors: contains datablock(s) I. DOI: 10.1107/S2414314620007920/hb4350Isup2.hkl

Supporting information file. DOI: 10.1107/S2414314620007920/hb4350Isup3.cml

CCDC reference: 2009305

Additional supporting information:  crystallographic information; 3D view; checkCIF report

## Figures and Tables

**Table 1:** Hydrogen-bond geometry (Å, °)

*D*—H⋯*A*	*D*—H	H⋯*A*	*D*⋯*A*	*D*—H⋯*A*
O2—H2⋯O1	0.84	1.86	2.586 (2)	144
C25—H25*A*⋯O2^i^	0.98	2.63	3.556 (3)	157
C28—H28*B*⋯O1^ii^	0.98	2.64	3.247 (3)	120

Symmetry codes: (i) 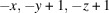 ; (ii) 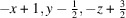 .

**Table 2:** Experimental details

Crystal data	
Chemical formula	C_28_H_28_O_6_
*M*_r_	460.50
Crystal system, space group	Monoclinic, *P*2_1_/*c*
Temperature (K)	200
*a*, *b*, *c* (Å)	9.9641 (9), 10.2338 (9), 23.541 (2)
β (°)	100.086 (2)
*V* (Å^3^)	2363.4 (4)
*Z*	4
Radiation type	Mo *K*α
μ (mm^−1^)	0.09
Crystal size (mm)	0.12 × 0.09 × 0.06

Data collection	
Diffractometer	Bruker APEXII CCD
No. of measured, independent and observed [*I* > 2σ(*I*)] reflections	14437, 4659, 2351
*R*_int_	0.058
(sin θ/λ)_max_ (Å^−1^)	0.617

Refinement	
*R*[*F* ^2^ > 2σ(*F* ^2^)], *wR*(*F* ^2^), *S*	0.046, 0.112, 0.88
No. of reflections	4659
No. of parameters	313
H-atom treatment	H-atom parameters constrained
Δρ_max_, Δρ_min_ (e Å^−3^)	0.20, −0.15

Computer programs: *APEX2* and *SAINT* (Bruker, 2012 ▸), *SHELXS* and *SHELXTL* (Sheldrick, 2008 ▸), *SHELXL2014* (Sheldrick, 2015 ▸) and *publCIF* (Westrip, 2010 ▸).

## full crystallographic data

### Crystal data

**Table:**

C_28_H_28_O_6_	*F*(000) = 976
*M**_r_* = 460.50	*D*_x_ = 1.294 Mg m^−^^3^
Monoclinic, *P*2_1_/*c*	Mo *K*α radiation, λ = 0.71073 Å
Hall symbol: -P 2ybc	Cell parameters from 3197 reflections
*a* = 9.9641 (9) Å	θ = 2.5–25.2°
*b* = 10.2338 (9) Å	µ = 0.09 mm^−^^1^
*c* = 23.541 (2) Å	*T* = 200 K
β = 100.086 (2)°	Block, orange
*V* = 2363.4 (4) Å^3^	0.12 × 0.09 × 0.06 mm
*Z* = 4	

### Data collection

**Table:**

Bruker APEXII CCD diffractometer	2351 reflections with *I* > 2σ(*I*)
Radiation source: fine-focus sealed tube	*R*_int_ = 0.058
Graphite monochromator	θ_max_ = 26.0°, θ_min_ = 1.8°
phi and ω scans	*h* = −11→12
14437 measured reflections	*k* = −9→12
4659 independent reflections	*l* = −28→29

### Refinement

**Table:**

Refinement on *F*^2^	Primary atom site location: structure-invariant direct methods
Least-squares matrix: full	Secondary atom site location: difference Fourier map
*R*[*F*^2^ > 2σ(*F*^2^)] = 0.046	Hydrogen site location: inferred from neighbouring sites
*wR*(*F*^2^) = 0.112	H-atom parameters constrained
*S* = 0.88	*w* = 1/[σ^2^(*F*_o_^2^) + (0.038*P*)^2^] where *P* = (*F*_o_^2^ + 2*F*_c_^2^)/3
4659 reflections	(Δ/σ)_max_ < 0.001
313 parameters	Δρ_max_ = 0.20 e Å^−^^3^
0 restraints	Δρ_min_ = −0.15 e Å^−^^3^

### Special details

**Table:**

Geometry. All esds (except the esd in the dihedral angle between two l.s. planes) are estimated using the full covariance matrix. The cell esds are taken into account individually in the estimation of esds in distances, angles and torsion angles; correlations between esds in cell parameters are only used when they are defined by crystal symmetry. An approximate (isotropic) treatment of cell esds is used for estimating esds involving l.s. planes.
Refinement. Refinement of F^2^ against ALL reflections. The weighted R-factor wR and goodness of fit S are based on F^2^, conventional R-factors R are based on F, with F set to zero for negative F^2^. The threshold expression of F^2^ > 2sigma(F^2^) is used only for calculating R-factors(gt) etc. and is not relevant to the choice of reflections for refinement. R-factors based on F^2^ are statistically about twice as large as those based on F, and R- factors based on ALL data will be even larger.

### Fractional atomic coordinates and isotropic or equivalent isotropic displacement parameters (Å^2^)

**Table:**

	*x*	*y*	*z*	*U*_iso_*/*U*_eq_	
C1	0.3422 (2)	0.3627 (2)	0.40004 (9)	0.0406 (6)	
C2	0.2593 (2)	0.4124 (2)	0.35097 (9)	0.0394 (6)	
C3	0.1584 (2)	0.5038 (2)	0.35435 (9)	0.0408 (6)	
H3	0.1026	0.5355	0.3203	0.049*	
C4	0.1399 (2)	0.5484 (2)	0.40788 (9)	0.0406 (6)	
C5	0.2191 (2)	0.5016 (2)	0.45928 (9)	0.0388 (6)	
C6	0.3192 (2)	0.4054 (2)	0.45383 (9)	0.0379 (5)	
C7	0.2088 (2)	0.5513 (2)	0.51624 (9)	0.0439 (6)	
H7	0.2752	0.5181	0.5468	0.053*	
C8	0.1215 (2)	0.6356 (2)	0.53234 (9)	0.0527 (7)	
H8	0.0514	0.6709	0.5039	0.063*	
C9	0.1295 (2)	0.6759 (2)	0.59251 (10)	0.0466 (6)	
O1	0.20637 (17)	0.62039 (17)	0.63223 (7)	0.0635 (5)	
C10	0.0400 (2)	0.7828 (2)	0.60542 (9)	0.0417 (6)	
C11	0.0227 (2)	0.8074 (2)	0.66249 (10)	0.0455 (6)	
O2	0.09471 (17)	0.74132 (18)	0.70846 (6)	0.0597 (5)	
H2	0.1521	0.6923	0.6970	0.090*	
C12	−0.0714 (2)	0.8998 (2)	0.67300 (11)	0.0528 (7)	
H12	−0.0858	0.9135	0.7114	0.063*	
C13	−0.1444 (2)	0.9722 (2)	0.62869 (11)	0.0533 (7)	
H13	−0.2084	1.0354	0.6367	0.064*	
C14	−0.1245 (2)	0.9527 (2)	0.57248 (11)	0.0508 (6)	
C15	−0.0335 (2)	0.8589 (2)	0.56154 (10)	0.0469 (6)	
H15	−0.0203	0.8455	0.5230	0.056*	
C16	0.4010 (2)	0.3476 (2)	0.50661 (9)	0.0422 (6)	
H16	0.4946	0.3709	0.5157	0.051*	
C17	0.3536 (2)	0.2669 (2)	0.54151 (9)	0.0459 (6)	
H17	0.2607	0.2422	0.5308	0.055*	
C18	0.4276 (2)	0.2103 (2)	0.59522 (9)	0.0408 (6)	
C19	0.5437 (2)	0.2690 (2)	0.62614 (9)	0.0433 (6)	
H19	0.5750	0.3489	0.6126	0.052*	
C20	0.6147 (2)	0.2142 (2)	0.67603 (9)	0.0439 (6)	
H20	0.6942	0.2559	0.6962	0.053*	
C21	0.5698 (2)	0.0981 (2)	0.69667 (9)	0.0405 (6)	
C22	0.4544 (2)	0.0388 (2)	0.66742 (9)	0.0434 (6)	
H22	0.4229	−0.0406	0.6814	0.052*	
C23	0.3837 (2)	0.0955 (2)	0.61703 (9)	0.0456 (6)	
H23	0.3035	0.0544	0.5972	0.055*	
C24	0.4492 (2)	0.2627 (2)	0.39366 (10)	0.0577 (7)	
H24A	0.4070	0.1896	0.3702	0.087*	
H24B	0.4913	0.2306	0.4319	0.087*	
H24C	0.5191	0.3028	0.3747	0.087*	
O3	0.28463 (16)	0.36458 (15)	0.29960 (6)	0.0508 (4)	
C25	0.1959 (2)	0.4029 (2)	0.24813 (9)	0.0553 (7)	
H25A	0.1021	0.3785	0.2507	0.083*	
H25B	0.2232	0.3589	0.2150	0.083*	
H25C	0.2014	0.4978	0.2433	0.083*	
O4	0.04512 (16)	0.64150 (15)	0.41344 (6)	0.0542 (5)	
C26	−0.0374 (3)	0.6922 (2)	0.36263 (10)	0.0626 (8)	
H26A	0.0211	0.7301	0.3375	0.094*	
H26B	−0.0980	0.7599	0.3733	0.094*	
H26C	−0.0921	0.6215	0.3422	0.094*	
O5	−0.18961 (18)	1.01996 (17)	0.52486 (7)	0.0685 (5)	
C27	−0.3001 (3)	1.1009 (3)	0.53203 (12)	0.0764 (9)	
H27A	−0.3682	1.0493	0.5476	0.115*	
H27B	−0.3415	1.1378	0.4946	0.115*	
H27C	−0.2673	1.1719	0.5589	0.115*	
O6	0.64798 (16)	0.05087 (16)	0.74612 (6)	0.0559 (5)	
C28	0.5936 (3)	−0.0556 (2)	0.77410 (10)	0.0634 (7)	
H28A	0.5010	−0.0341	0.7797	0.095*	
H28B	0.6513	−0.0717	0.8116	0.095*	
H28C	0.5911	−0.1342	0.7501	0.095*	

### Atomic displacement parameters (Å^2^)

**Table:**

	*U*^11^	*U*^22^	*U*^33^	*U*^12^	*U*^13^	*U*^23^
C1	0.0444 (14)	0.0374 (13)	0.0385 (13)	0.0065 (11)	0.0035 (11)	−0.0028 (11)
C2	0.0446 (14)	0.0410 (14)	0.0322 (13)	−0.0025 (12)	0.0057 (11)	−0.0050 (11)
C3	0.0465 (14)	0.0433 (14)	0.0301 (13)	0.0046 (12)	−0.0003 (11)	0.0010 (10)
C4	0.0431 (14)	0.0390 (14)	0.0383 (14)	0.0096 (11)	0.0033 (11)	0.0007 (11)
C5	0.0443 (14)	0.0409 (14)	0.0297 (13)	0.0038 (11)	0.0020 (11)	−0.0007 (10)
C6	0.0420 (13)	0.0368 (13)	0.0332 (13)	0.0038 (11)	0.0024 (11)	0.0013 (10)
C7	0.0517 (15)	0.0453 (14)	0.0322 (13)	0.0119 (12)	0.0001 (11)	−0.0005 (11)
C8	0.0569 (16)	0.0656 (17)	0.0331 (14)	0.0220 (14)	0.0013 (12)	−0.0053 (12)
C9	0.0468 (15)	0.0571 (16)	0.0346 (14)	0.0073 (13)	0.0036 (12)	−0.0024 (12)
O1	0.0732 (12)	0.0785 (13)	0.0345 (10)	0.0290 (10)	−0.0025 (9)	−0.0017 (9)
C10	0.0405 (14)	0.0487 (15)	0.0354 (13)	0.0027 (12)	0.0055 (11)	−0.0061 (11)
C11	0.0477 (15)	0.0524 (16)	0.0357 (14)	−0.0025 (13)	0.0051 (12)	−0.0064 (12)
O2	0.0653 (12)	0.0778 (13)	0.0347 (9)	0.0115 (10)	0.0050 (9)	−0.0063 (9)
C12	0.0548 (16)	0.0628 (17)	0.0435 (15)	−0.0002 (14)	0.0158 (13)	−0.0153 (13)
C13	0.0513 (16)	0.0538 (17)	0.0581 (17)	0.0032 (13)	0.0183 (14)	−0.0120 (14)
C14	0.0493 (15)	0.0523 (16)	0.0510 (16)	0.0082 (13)	0.0096 (13)	−0.0006 (13)
C15	0.0480 (15)	0.0552 (16)	0.0384 (14)	0.0066 (13)	0.0098 (12)	−0.0046 (12)
C16	0.0397 (13)	0.0439 (14)	0.0403 (14)	0.0059 (11)	−0.0001 (11)	0.0023 (11)
C17	0.0457 (15)	0.0514 (15)	0.0381 (14)	0.0013 (12)	0.0005 (12)	0.0032 (12)
C18	0.0475 (14)	0.0403 (14)	0.0336 (13)	0.0047 (12)	0.0045 (11)	0.0001 (11)
C19	0.0529 (15)	0.0392 (14)	0.0375 (14)	0.0033 (12)	0.0067 (12)	−0.0002 (11)
C20	0.0473 (15)	0.0466 (15)	0.0354 (14)	0.0008 (12)	0.0008 (12)	0.0001 (11)
C21	0.0453 (14)	0.0450 (15)	0.0306 (13)	0.0069 (12)	0.0055 (11)	0.0023 (11)
C22	0.0498 (15)	0.0430 (14)	0.0390 (14)	0.0038 (12)	0.0126 (12)	0.0045 (12)
C23	0.0459 (14)	0.0498 (15)	0.0405 (14)	0.0018 (12)	0.0057 (12)	−0.0002 (12)
C24	0.0645 (18)	0.0603 (17)	0.0475 (16)	0.0204 (14)	0.0074 (14)	−0.0043 (13)
O3	0.0576 (11)	0.0620 (11)	0.0324 (9)	0.0061 (9)	0.0071 (8)	−0.0083 (8)
C25	0.0583 (16)	0.0779 (19)	0.0280 (13)	−0.0043 (14)	0.0028 (12)	−0.0054 (13)
O4	0.0621 (11)	0.0618 (11)	0.0352 (9)	0.0287 (9)	−0.0016 (8)	0.0006 (8)
C26	0.0670 (18)	0.0717 (19)	0.0444 (16)	0.0338 (15)	−0.0030 (13)	0.0097 (14)
O5	0.0725 (13)	0.0722 (13)	0.0627 (12)	0.0316 (11)	0.0176 (10)	0.0101 (10)
C27	0.0694 (19)	0.073 (2)	0.087 (2)	0.0320 (17)	0.0160 (17)	0.0081 (17)
O6	0.0590 (11)	0.0652 (12)	0.0398 (10)	0.0011 (9)	−0.0013 (8)	0.0186 (9)
C28	0.0746 (19)	0.0636 (18)	0.0492 (16)	−0.0003 (15)	0.0034 (14)	0.0242 (14)

### Geometric parameters (Å, º)

**Table:**

C1—C2	1.393 (3)	C17—C18	1.467 (3)
C1—C6	1.396 (3)	C17—H17	0.9500
C1—C24	1.504 (3)	C18—C23	1.383 (3)
C2—O3	1.368 (2)	C18—C19	1.391 (3)
C2—C3	1.386 (3)	C19—C20	1.379 (3)
C3—C4	1.383 (3)	C19—H19	0.9500
C3—H3	0.9500	C20—C21	1.388 (3)
C4—O4	1.364 (2)	C20—H20	0.9500
C4—C5	1.408 (3)	C21—O6	1.370 (2)
C5—C6	1.423 (3)	C21—C22	1.374 (3)
C5—C7	1.454 (3)	C22—C23	1.396 (3)
C6—C16	1.484 (3)	C22—H22	0.9500
C7—C8	1.327 (3)	C23—H23	0.9500
C7—H7	0.9500	C24—H24A	0.9800
C8—C9	1.464 (3)	C24—H24B	0.9800
C8—H8	0.9500	C24—H24C	0.9800
C9—O1	1.238 (3)	O3—C25	1.424 (2)
C9—O1	1.238 (3)	C25—H25A	0.9800
C9—C10	1.476 (3)	C25—H25B	0.9800
C10—C15	1.395 (3)	C25—H25C	0.9800
C10—C11	1.407 (3)	O4—C26	1.425 (2)
C11—O2	1.368 (3)	C26—H26A	0.9800
C11—C12	1.384 (3)	C26—H26B	0.9800
O2—H2	0.8400	C26—H26C	0.9800
C12—C13	1.379 (3)	O5—C27	1.411 (3)
C12—H12	0.9500	C27—H27A	0.9800
C13—C14	1.386 (3)	C27—H27B	0.9800
C13—H13	0.9500	C27—H27C	0.9800
C14—C15	1.376 (3)	O6—C28	1.429 (3)
C14—O5	1.377 (3)	C28—H28A	0.9800
C15—H15	0.9500	C28—H28B	0.9800
C16—C17	1.310 (3)	C28—H28C	0.9800
C16—H16	0.9500		
			
C2—C1—C6	118.1 (2)	C18—C17—H17	116.3
C2—C1—C24	119.5 (2)	C23—C18—C19	117.4 (2)
C6—C1—C24	122.4 (2)	C23—C18—C17	120.8 (2)
O3—C2—C3	122.7 (2)	C19—C18—C17	121.8 (2)
O3—C2—C1	115.3 (2)	C20—C19—C18	121.7 (2)
C3—C2—C1	122.0 (2)	C20—C19—H19	119.2
C4—C3—C2	119.3 (2)	C18—C19—H19	119.2
C4—C3—H3	120.3	C19—C20—C21	119.8 (2)
C2—C3—H3	120.3	C19—C20—H20	120.1
O4—C4—C3	121.6 (2)	C21—C20—H20	120.1
O4—C4—C5	116.66 (19)	O6—C21—C22	124.9 (2)
C3—C4—C5	121.8 (2)	O6—C21—C20	115.4 (2)
C4—C5—C6	117.02 (19)	C22—C21—C20	119.7 (2)
C4—C5—C7	123.7 (2)	C21—C22—C23	119.7 (2)
C6—C5—C7	119.2 (2)	C21—C22—H22	120.1
C1—C6—C5	121.8 (2)	C23—C22—H22	120.1
C1—C6—C16	118.8 (2)	C18—C23—C22	121.6 (2)
C5—C6—C16	119.41 (19)	C18—C23—H23	119.2
C8—C7—C5	130.2 (2)	C22—C23—H23	119.2
C8—C7—H7	114.9	C1—C24—H24A	109.5
C5—C7—H7	114.9	C1—C24—H24B	109.5
C7—C8—C9	122.2 (2)	H24A—C24—H24B	109.5
C7—C8—H8	118.9	C1—C24—H24C	109.5
C9—C8—H8	118.9	H24A—C24—H24C	109.5
O1—C9—C8	121.5 (2)	H24B—C24—H24C	109.5
O1—C9—C8	121.5 (2)	C2—O3—C25	118.06 (17)
O1—C9—C10	120.0 (2)	O3—C25—H25A	109.5
O1—C9—C10	120.0 (2)	O3—C25—H25B	109.5
C8—C9—C10	118.4 (2)	H25A—C25—H25B	109.5
C15—C10—C11	118.1 (2)	O3—C25—H25C	109.5
C15—C10—C9	121.3 (2)	H25A—C25—H25C	109.5
C11—C10—C9	120.6 (2)	H25B—C25—H25C	109.5
O2—C11—C12	118.3 (2)	C4—O4—C26	118.78 (17)
O2—C11—C10	122.2 (2)	O4—C26—H26A	109.5
C12—C11—C10	119.5 (2)	O4—C26—H26B	109.5
C11—O2—H2	109.5	H26A—C26—H26B	109.5
C13—C12—C11	121.1 (2)	O4—C26—H26C	109.5
C13—C12—H12	119.5	H26A—C26—H26C	109.5
C11—C12—H12	119.5	H26B—C26—H26C	109.5
C12—C13—C14	120.0 (2)	C14—O5—C27	117.51 (19)
C12—C13—H13	120.0	O5—C27—H27A	109.5
C14—C13—H13	120.0	O5—C27—H27B	109.5
C15—C14—O5	115.4 (2)	H27A—C27—H27B	109.5
C15—C14—C13	119.2 (2)	O5—C27—H27C	109.5
O5—C14—C13	125.4 (2)	H27A—C27—H27C	109.5
C14—C15—C10	121.9 (2)	H27B—C27—H27C	109.5
C14—C15—H15	119.0	C21—O6—C28	117.08 (18)
C10—C15—H15	119.0	O6—C28—H28A	109.5
C17—C16—C6	124.8 (2)	O6—C28—H28B	109.5
C17—C16—H16	117.6	H28A—C28—H28B	109.5
C6—C16—H16	117.6	O6—C28—H28C	109.5
C16—C17—C18	127.4 (2)	H28A—C28—H28C	109.5
C16—C17—H17	116.3	H28B—C28—H28C	109.5
			
C6—C1—C2—O3	−178.50 (18)	C9—C10—C11—O2	5.1 (3)
C24—C1—C2—O3	−0.5 (3)	C15—C10—C11—C12	3.7 (3)
C6—C1—C2—C3	1.5 (3)	C9—C10—C11—C12	−173.9 (2)
C24—C1—C2—C3	179.5 (2)	O2—C11—C12—C13	178.2 (2)
O3—C2—C3—C4	−179.4 (2)	C10—C11—C12—C13	−2.8 (4)
C1—C2—C3—C4	0.6 (3)	C11—C12—C13—C14	0.2 (4)
C2—C3—C4—O4	177.77 (19)	C12—C13—C14—C15	1.3 (4)
C2—C3—C4—C5	−1.2 (3)	C12—C13—C14—O5	−179.0 (2)
O4—C4—C5—C6	−179.35 (18)	O5—C14—C15—C10	−179.9 (2)
C3—C4—C5—C6	−0.3 (3)	C13—C14—C15—C10	−0.2 (4)
O4—C4—C5—C7	−2.9 (3)	C11—C10—C15—C14	−2.3 (3)
C3—C4—C5—C7	176.1 (2)	C9—C10—C15—C14	175.4 (2)
C2—C1—C6—C5	−3.1 (3)	C1—C6—C16—C17	−108.8 (3)
C24—C1—C6—C5	179.0 (2)	C5—C6—C16—C17	70.8 (3)
C2—C1—C6—C16	176.4 (2)	C6—C16—C17—C18	−177.6 (2)
C24—C1—C6—C16	−1.5 (3)	C16—C17—C18—C23	−157.3 (2)
C4—C5—C6—C1	2.5 (3)	C16—C17—C18—C19	22.7 (4)
C7—C5—C6—C1	−174.1 (2)	C23—C18—C19—C20	1.3 (3)
C4—C5—C6—C16	−177.0 (2)	C17—C18—C19—C20	−178.7 (2)
C7—C5—C6—C16	6.4 (3)	C18—C19—C20—C21	−0.5 (3)
C4—C5—C7—C8	6.9 (4)	C19—C20—C21—O6	179.34 (19)
C6—C5—C7—C8	−176.8 (2)	C19—C20—C21—C22	−0.4 (3)
C5—C7—C8—C9	−178.8 (2)	O6—C21—C22—C23	−179.4 (2)
C7—C8—C9—O1	−9.8 (4)	C20—C21—C22—C23	0.3 (3)
C7—C8—C9—O1	−9.8 (4)	C19—C18—C23—C22	−1.4 (3)
C7—C8—C9—C10	172.0 (2)	C17—C18—C23—C22	178.6 (2)
C8—C9—O1—O1	0.0 (3)	C21—C22—C23—C18	0.6 (3)
C10—C9—O1—O1	0.0 (3)	C3—C2—O3—C25	−5.3 (3)
O1—C9—C10—C15	171.1 (2)	C1—C2—O3—C25	174.72 (19)
O1—C9—C10—C15	171.1 (2)	C3—C4—O4—C26	0.8 (3)
C8—C9—C10—C15	−10.8 (3)	C5—C4—O4—C26	179.9 (2)
O1—C9—C10—C11	−11.4 (3)	C15—C14—O5—C27	169.4 (2)
O1—C9—C10—C11	−11.4 (3)	C13—C14—O5—C27	−10.2 (4)
C8—C9—C10—C11	166.8 (2)	C22—C21—O6—C28	−10.6 (3)
C15—C10—C11—O2	−177.2 (2)	C20—C21—O6—C28	169.73 (19)

### Hydrogen-bond geometry (Å, º)

**Table:**

*D*—H···*A*	*D*—H	H···*A*	*D*···*A*	*D*—H···*A*
O2—H2···O1	0.84	1.86	2.586 (2)	144
C25—H25*A*···O2^i^	0.98	2.63	3.556 (3)	157
C28—H28*B*···O1^ii^	0.98	2.64	3.247 (3)	120

Symmetry codes: (i) −*x*, −*y*+1, −*z*+1; (ii) −*x*+1, *y*−1/2, −*z*+3/2.
